# Concomitant elevated serum levels of tenascin, MMP-9 and YKL-40, suggest ongoing remodeling of the heart up to 3 months after cardiac surgery after normalization of the revascularization markers

**DOI:** 10.1186/s40001-022-00831-8

**Published:** 2022-10-21

**Authors:** Da Liu, Danyal Ghani, Justin Wain, Wilson Y. Szeto, Krzysztof Laudanski

**Affiliations:** 1 Department of Obstetrics and Gynecology, Shengjing Hospital of China Medical University, Shenyang, People’s Republic of China; 2 College of Art and Sciences, Drexel University, Philadelphia, PA USA; 3 Campbell University School of Osteopathic Medicine, Buies Creek, NC USA; 4 Department of Cardiac Surgery, University of Pennsylvania, Philadelphia, PA USA; 5 Department of Anesthesiology and Critical Care, University of Pennsylvania, Philadelphia, PA USA; 6 Department of Neurology, University of Pennsylvania, Philadelphia, PA USA; 7 Leonard Davis Institute for Health Economics, University of Pennsylvania, JMB 127, 3620 Hamilton Walk, Philadelphia, PA 19146 USA

**Keywords:** Cardiac surgery, HSP-70, IL-8, C-reactive protein, VEGF, MCP-1, Epiregulin, Tenascin, YKL-40, MMP-9, Long-term, Disposition

## Abstract

**Background:**

The recovery from cardiac surgery involves resolving inflammation and remodeling with significant connective tissue turnover. Dynamics of smoldering inflammation and injury (white blood cells, platelets, CRP, IL-8, IL-6), vascular inflammation (IL-15, VEGF, RANTES), connective tissue remodeling (tenascin, MMP-9), cardiac injury and remodeling (YKL-40), and vascular remodeling (epiregulin, MCP-1, VEGF) were assessed up to 3 months after cardiac surgery. We hypothesize that at 3 months, studied markers will return to pre-surgical levels.

**Methods:**

Patients (n = 139) scheduled for non-emergent heart surgery were included, except for patients with pre-existing immunological aberrancies. Blood was collected before surgery(t_baseline_), 24 h later(t_24h_) after the first sample, 7 days(t_7d_), and 3 months(t_3m_) after t_baseline_. Serum markers were measured via multiplex or ELISA. Electronic medical records (EMR) were used to extract demographical, pre-existing conditions and clinical data. Disposition (discharge home, discharge to facility, death, re-admission) was determined at 28 days and 3 months from admission.

**Results:**

Not all inflammatory markers returned to baseline (CRP↑↑, leukocytosis, thrombocytosis, IL-8↓, IL-6↓). Tenascin and YKL-40 levels remained elevated even at t_3m_. YKL-40 serum levels were significantly elevated at t_24h_ and t_7d_ while normalized at t_3m_. VEGF returned to the baseline, yet MCP-1 remained elevated at 3 months. CCL28 increased at 3 months, while RANTES and IL-15 declined at the same time. Disposition at discharge was determined by serum MMP-9, while YKL-40 correlated with duration of surgery and APACHE II_24h_.

**Conclusions:**

The data demonstrated an ongoing extracellular matrix turnover at 3 months, while acute inflammation and vascular remodeling resolved only partially.

**Supplementary Information:**

The online version contains supplementary material available at 10.1186/s40001-022-00831-8.

## Background

Cardiac surgery triggers an intense inflammatory process [1–4]. Therefore, for optimal outcome, acute inflammation has to be resolved to provide the foundation for tissue healing and remodeling [4–7]. However, the time extent of the inflammation and subsequent remodeling in the wake of cardiac surgery is unknown [4, 6]. Determining the duration of inflammation and remodeling process is essential to establish when homeostasis and tissue repair recovery is complete after cardiac surgery. Conversely, the unfavorable resolution of inflammation may result in worsening vascular flow by increased vascular wall stiffness, progression of atherosclerosis, and vascular dysfunction [8–12]. All these conditions limit the long-term benefit of heart surgery [4, 7].

Apart from mechanical and free radical stress, the physical dissection of the tissue needs to be executed to gain access to the structure in need of repair [13, 14]. Then, almost all surgeries involve a mechanical intervention into the physical integrity of the myocardium and blood supply to the heart [2]. At a minimum, the sternum is dissected, and the coronary arteries are opened and anastomosed with grafted arteries and/or veins during the coronary arteries bypass graft (CABG) surgery [2]. CABG is often accompanied by other procedures (Cox maze procedure, valve repair, others), which are even more inflammatory and destructive to cardiac tissue [2]. Theoretically, the extent of the insult should vary between different cardiac surgery types [2, 15]. Consequently, blood vessels are subjected to significant inflammation secondary to the iatrogenic injury, while perivascular adipose tissue moderates this process [16, 17]. Vascular inflammation impairs tissue healing after surgery and is an independent factor in the progression of cardiovascular disease [18, 19]. This will limit the benefit of the surgery aimed at improving coronary flow and oxygen delivery to the myocardium [20, 21]. The damaged tissues release several danger-associated patterns (DAMPs) secondary to the damaged extracellular matrix (heparan, fibronectin, tenascin). In contrast, others are primarily intracellular molecules released secondary to the physical destruction of the cell (ADAM17, high mobility box protein—1, heat shock proteins, histones, DNA, RNA, mtDNA, S100) [15, 22–27]. DAMPs release is a potent activator of the immune system and subsequent mortality, but at the same time, it is critical in activating repair processes [15, 24, 25]. The repair process is driven by the immune system and local regenerative mechanisms and involves rebuilding the extracellular matrix and tissue structure, with revascularization being the critical part [5, 28]. Matrix metalloproteinase 9 (MMP-9) is critical in rebuilding the tissue and revascularization. However, abnormal MMP-9 expression is linked to the emergence of atrial fibrillation and aortic aneurysm by faulty remodeling of cardiovascular tissue and/or affecting neutrophile activation and chemotaxis [25, 26, 29–33]. Other extracellular protein-like YKL-40 and tenascin have a similar role, with turnover increased during damage, remodeling, and mechanical stress to the cardiac tissue. The process of remodeling the cardiac tissue after surgery is accompanied by intense revascularization and vascular inflammation [28]. The revascularization process is complex and driven by the local tissue environment, monocytes, and several cytokines with VEGF, Tie-2, FGF-2, GM-CSF, MCP-1, epiregulin, Ang-1, and platelets playing the essential function [18, 34–36]. GM-CSF, TGFβ, and MCP-1 have a particularly synergistic relation for arteriogenesis, a necessary process for building collateral blood vessels after ischemia [6, 24, 35, 37, 38]. Their mechanisms of augmenting collateral flow include monocyte activation and stimulation of TNFα, IL-1β, and FGF-2 [39]. Angiogenesis stimulation factors and clearance of the damaged tissue are enabling factors for this process [5, 6, 24, 34].

Successful resolution of the post-surgical inflammation, tissue remodeling, and revascularization augments recovery [4]. An inappropriate milieu of the endothelial vascular inflammation markers will result in unfavorable recovery and premature failure of grafted blood vessels [7, 16, 19–21, 40]. Furthermore, TGFβ-, ADAM-17-, and MMP-9-induced fibrosis emerge, resulting in decompensated oxygen delivery, increased myocardial stiffness, aneurysmal characteristics, and progression of atherosclerosis [5, 27, 37, 41–44]. Several of these markers are actively released during cardiac surgery, but the duration and magnitude of their release have not been determined in a study extending long-term after cardiac surgery [38]. Preliminary data demonstrated the abnormal function of monocytes (MO), critical cells for vascularization and arteriogenesis processes, is impaired for 3 months after surgery, suggesting an increased and protracted risk of unfavorable revascularization [45–47]. Finally, thrombocytopenia has been suggested a risk factor for adverse effect during cardiac surgery [40].

Though some reports addressed the dynamic of arterio- and atherosclerosis markers after cardiac surgery, the observation is limited to the acute perioperative period [18, 19, 21, 35, 48]. In addition, measurements are most commonly focused on one factor, while the process of post-surgical recovery is determined by a milieu of the DAMP, inflammatory response, anti-inflammatory factors, and cytokines supporting the process of arteriogenesis and tissue repair [18–20, 28, 35, 49, 50]. There is a gap in knowledge when the remodeling milieu returns to the pre-surgical level compared to the inflammatory process's resolution. Determining such a period is essential and would enable to find a physiological, not arbitrary, reference point to evaluate the outcome of cardiac surgery. Consequently, in this research, we hypothesize that serum DAMP (Hsp-60), resolution of inflammation (CRP, IL-6, IL-8), vascular remodeling (VEGF, MCP-1, epiregulin), and tissue repair (tenascin, YKL-40, MMP-9) markers will return to pre-surgical after 3 months from the cardiac surgery [1, 7, 16, 33, 36, 51, 52]. In addition, we examined the serum level of IL-15, CCL28, and RANTES as the critical indicators of vascular inflammation [51, 53–55]. The persistence of some of these markers (MMP-9, CRP, NT-BNP, IL-15, RANTES, CCL28) may indicate unfavorable resolution of the inflammatory process [16, 17, 33, 51, 53, 56–60]. Clinically, these markers would indicate a potentially increased risk of postoperative fibrosis or atrial fibrillation [25, 32, 33, 56, 61].

## Methods

### Patients enrollment

#### Our study protocol was approved by the Institutional Review Board (IRB) (#815686)

All patients scheduled for non-emergent heart surgery were approached for consent. We excluded patients with pre-existing immunological aberrancies who were on immunosuppressant medications in the last 6 months (prednisone PO or IV more than 5 mg daily, αTNFα, αIL-6, αIL-3, αCD20 antibodies therapy, immunoglobulin, plasmapheresis, methotrexate, chemotherapy). The study did not include patients with known inherited dyslipidemias and post-transplant.

Consent was done before surgery in the condition following the procedure to secure consent by surgical staff. Consent was sought from a patient or their surrogate. The study did not include minors.

The demographic characteristic of the studied individuals is presented in Table 1.

**Table 1:** Patient characteristics

Patient characteristics (*N* = 139)	
Age, mean ± SD [years]	64.03 ± 12.69
Sex—Male no (% of total)	104 (74.8%)
BMI mean ± SD [kg/m^2^]	28.6 ± 6.03
Race (%Caucasian, %Black % Asian % Other)	83.5%, 6.5%, 3.6%, 6.5%
Anesthesia and surgery data	
Duration of anesthesia; mean ± SD [min]	393.81 ± 119.41
Duration of surgery; mean ± SD [min]	277.96 ± 102.86
Coronary artery bypass surgery^a^; no	54
Mitral valvuloplasty and replacement^a^; no	16
Aortic valvuloplasty and replacement^a^; no	25
Aortic aneurysm repair^a^; no	5
Others^a^; no	0
Perioperative management	
Estimated blood loss [ml]	209.49 ± 286.92
Total crystalloid during surgery [ml]	1263.01 ± 611.93
Corticosteroid administration (% of all cases)	10.1%
Ketorolac administration (% of all cases)	7.9%
Acetaminophen administration (% of all cases)	73.4%
ASA administration	66.9%
ICU stay	
APACHE score at 1 h, mean ± SD	16.86 ± 6.03
APACHE score at 24 h, mean ± SD	9.73 ± 4.96
APACHE score at 48 h, mean ± SD	9.25 ± 4.78
Comorbidities	
CCI median (95%CI)	4.04 ± 2.27
Acute coronary syndrome	13.7%
Congestive heart failure	21.6%
Peripheral vascular disease	9.4%
Cerebrovascular accident	10.1%
Chronic obstructive pulmonary disease	7.2%
Connective tissue disease (non-active)	17.3%
Peptic ulcer disease	7.2%
Mild liver disease	5%
Type 2 diabetes	29.5%
Renal disease	29.5%
Diabetes with organ damage	1.4%
Tumor	8.6%
Outcomes	
Mortality	3.6% Dead
CVA	4.3% Before surgery, 6.5% After Surgery, 2.9% Both
PE	5% Before surgery
DVT	5.8% Before surgery, 2.9% After

^a^Some patients had more than one procedure

#### Sample processing

Upon consent, blood was collected in vacutainer tubes with heparin, cooled, and spun down. Serum was isolated and stored at − 80 °C. Blood was collected four times. T_baseline_ sample was collected before or shortly after arterial or central line placement(t_baseline_). t_24h_ was collected during patient’s stay in the ICU, 24 h after the first sample. Second, t_7d_ specimen was obtained at the patient's discharge from the hospital or 7 days after t_baseline_. Finally, the last sample was collected no sooner than 3 months after surgery but no later than 4 months (t_3m_). The sampling represents baseline, acute stress response, convalescence, and medium-term recovery.

#### Clinical data

The electronic medical records (EMR) were used to collect the demographic and clinical data for all the enrolled participants. Patients self-determined race and ethnicity. Several variables regarding the duration of surgery and anesthesia were collected from medical records retrospectively. Preoperative Hb1ac and lipid profile was collected from routine pre-op labs when available. The Acute Physiology and Chronic Health Evaluation II (APACHE II) score was calculated within 1 h (APACHE_1h_) and at 24 (APACHE_24h_) and 48 h (APACHE_48h_) after admission to the ICU [62, 63]. The burden of chronic disease was calculated using the Charlson Comorbidity Index (CCI) [62, 64]. Disposition and survival were determined at 28 days from admission. White blood cell count (WBC) and platelet estimate were pulled from EHR as a part of routine medical care.

#### Assessment of biomarkers

MMP-9 and MCP-1, were collected using the ELISA technique according to manufacturer recommendations (BioLegend, San Diego, CA) [33, 65]. In addition, inflammatory markers (IL-6, CRP, IL-8) cardiac injury and remodeling markers (tenascin YKL-40), vascular inflammation (CCL28, RANTES, IL-15), and remodelling (epiregulin, VEGF) and were collected using multiplex technology (Theromofisher, Waltham, MA) on a MagPix machine (Luminex; Austin, TX) [52, 66–69].

#### Statistical Analysis

The Shapiro–Wilk *W* test and distribution plots were used to test the normality of distribution variables. Parametric variables were expressed as mean ± SD and compared using t-Student (t[n]). For non-parametric variables, median rank and 95% confidence interval (Me;CI95%) were used with the Mann–Whitney *U* (U[n]) statistic employed to compare such variables. ANOVA was calculated for parametric variables (F[df;n]) with multiple discrete values with Geisser–Greenhouse correction in the mixed model for missing data with Duncanc’s test as a post hoc test employed to determine the difference between groups. Kruskal Wallis test (KS[df;n]) was utilized in the case of non-parametric variables. Longitudinal and pairwise analysis was done in most of the statistical contrasts. Correlation momentums were calculated as *r* Pearson. The regression analysis was done using stepwise methods. A *p* value less than 0.05 was considered statistically significant for all tests. Statistical analyses were performed with SPSS 26 (IBM, Waltham, NY).

## Results

### Markes of tissue remodeling (tenascin, MMP-9; YKL-40) suggests an ongoing process of repair even 3 months after cardiac surgery

Gender and race did not affect the preoperative levels of tenascin, YKL-40, and MMP-9. Only in case of patients over 60, elevated level of YKL-40_t24_ (YKL-40_ovoer60_ = 41.87 ± 116.74 vs. YKL-40_below60_ = 60.23 ± 116.74; U[92] = 527; *p* = 0.003) was seen and YKL-40_t7d_ (YKL-40_ovoer60_ = 38.13 ± 98.29 vs. YKL-40_below60_ = 54.82 ± 98.29; U[83] = 411; *p* = 0.006). Neither CCI score nor the incidence of pre-existing conditions defined in that score did not differentiate patients. Correlation between HB1ac, NT-BNP, BMI, and pre-surgical troponin was low and not-significant.

**Fig. 1:**
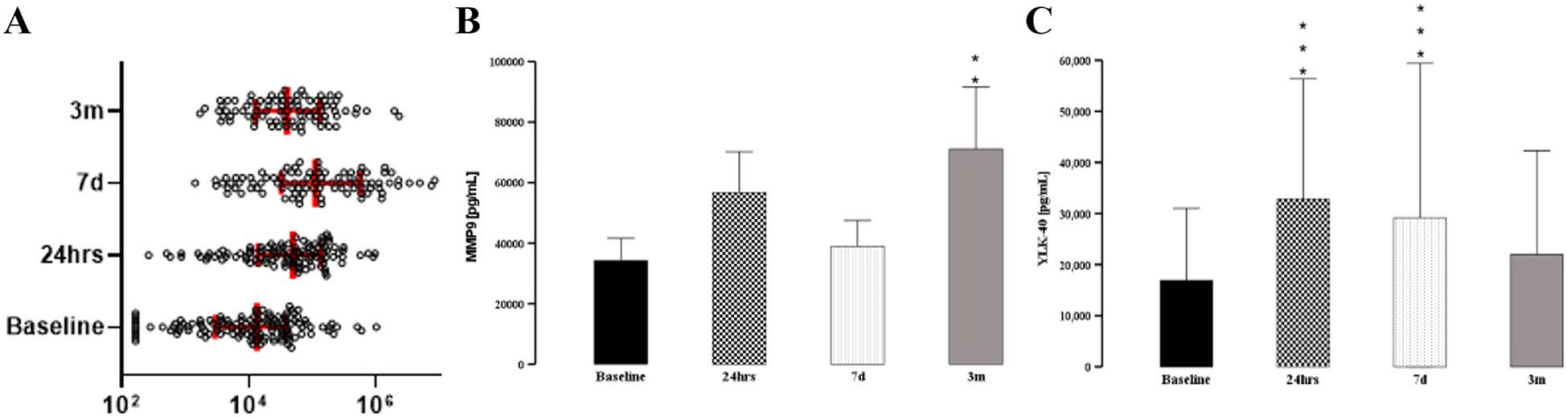
Evolution of the markers for tissue turnover after cardiac surgery. Serum tenascin level peaked at 7 days **A** after surgery. In contrast MMP-9 had the highest increase at 3 months **B,** while YKL-40 was transiently elevated at 24 h and 7 days **C**. * denotes *p* < 0.05, **p < 0.01, ***p < 0.001

Tenascin levels increase significantly after surgery as compared to pre-surgical levels, with the peak at 7 days (Z[94] = − 7.891; *p* =  < 0.001) and remaining elevated at 3 months (Z[85] = − 5.755; *p* =  < 0.001) (Fig. 1A). The increase at 7 days was more pronounced in female patients (tenascin_7dMale_ = 44.76 ± 116.76 vs. tannascin_7dFemale_ = 57.58 ± 116.76; U[95] = 1082; *p* = 0.049), but regression analysis did not demonstrate that sex is a significant predictor of tenascin serum level before surgery (β = 0.82; *p* = 0.368) and at any subsequent time point. Neither CCI, age, nor race was significant predictors of tenascin at t_24h_, t_7d_, and t_3m_.

Serum level of MMP-9 steadily increased after surgery as compared to pre-surgical levels, with 3 month follow-up demonstrating statistically significant change (Fig. 1B). Neither CCI, age, nor race were significant predictors of MMP-9 at t_24h_, t_7d_, and t_3m_.

YKL-40 serum levels were significantly elevated at 24 h and 7 days and normalized at 3 months, but very significant variability was observed at the last follow-up (Fig. 3C). Interestingly younger patients had a significantly higher level of YKL-40 at t_24h_ (YKL-40_ovoer60_ = 41.87 ± 116.74 vs. YKL-40_below60_ = 60.23 ± 116.74; U[93] = 527;*p* = 0.003) and t_7d_ (YKL-40_ovoer60_ = 38.13 ± 98.29 vs. YKL-40_below60_ = 54.82 ± 98.29; U[84] = 411; *p* = 0.006). Neither CCI, age, nor race were significant predictors of tenascin at t_24h_, t_7d_, and t_3m_ (Fig. 1C).

### Markers of vascular revascularization (MCP-1, epiregulin, VEGF) and inflammation (CCL28, RANTES, IL-15) demonstrated a complex picture

**Fig. 2:**
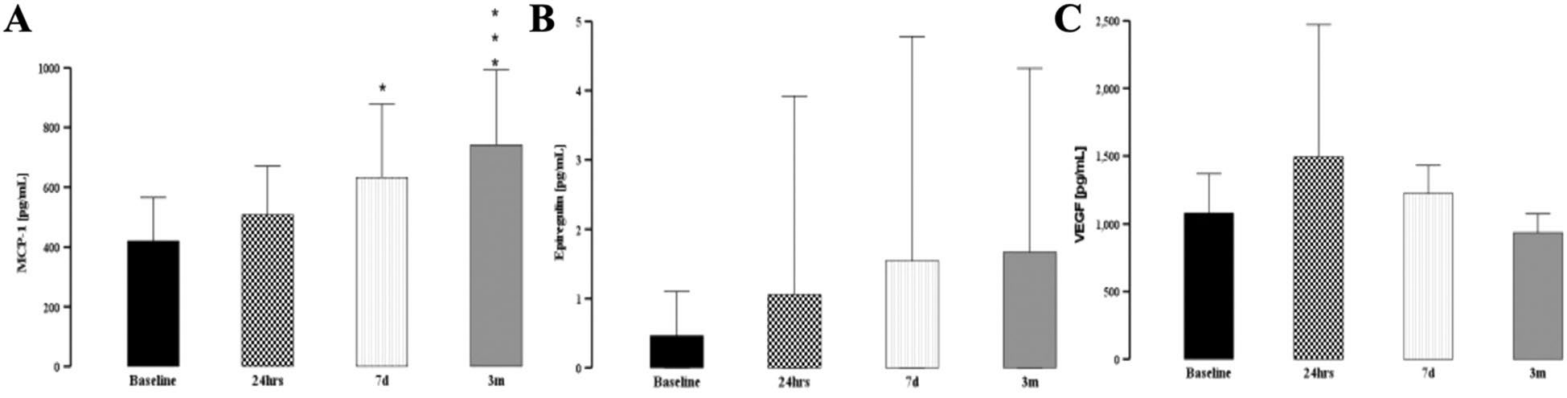
Evolution of revascularization markers for tissue turnover after cardiac surgery. Serum MCP-1 continue to increase after cardiac surgery at 7 days and 3 months **A** after surgery. Epiregulin levels were highly variable **B,** while VEGF post-operative variability did not reach the level of statisticl significance **C**. * denotes *p* < 0.05, **p < 0.01, ***p < 0.001

Markes of revascularization (MCP-1, epiregulin, and VEGF) had very heterogenous dynamics after cardiac surgery. Sex, race, and age did not determine MCP-1, VEGF, and epiregulin baseline levels (data not shown). Epiregulin levels tended to be elevated during recovery, but this studied variable was highly variable and statistically non-significant (data not shown). In contrast, serum VEGF increased peri-operatively to normalize to t_7d_ and t_3m_ (Fig. 2B).

RANTES, IL-15, and CCL28 before surgery did not differentiate patients in terms of gender, race, and age. RANTES in serum was elevated in patients with diabetes (U[20] = 62; *p* = 0.033) and correlated highly with Hb1ac (*r* = 0.7; *p* < 0.001). Pre-surgical level of CCL28 was affected by the history of stroke, peripheral vascular disease, and pre-existing mixed tissue autoimmune disease (Additional file 1: Figure S1). These differences persisted at 7 day and 3 month follow-up (data not shown). CCL28 correlated with NT-BNP at baseline (r = 0.61; *p* = 0.015) and with pre-surgical troponin levels at t_0_ (*r* =  0.83; *p* < 0.001), t_24h_ (*r* = 0.4; *p* = 0.015), and t_3m_ (*r* = 0.58; *p* = 0.004).

**Fig. 3:**
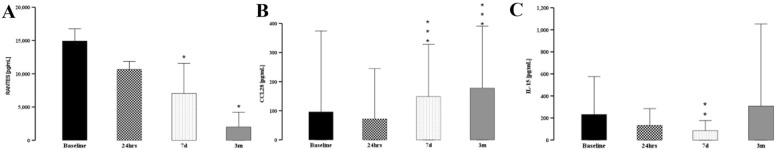
Evolution of vascular inflammation after cardiac surgery. Serum RANTES declined after cardiac surgery with nadir at 3 months **A**. Conversely, CCL-28 increased post-operatively at 7 days and 3 months **B**. IL-15 had tenedncy to be diminished in immidiate perioperative period with recovery to pre-operative baseline at 3 months **C**. * denotes *p* < 0.05, **p < 0.01, ***p < 0.001

RANTES serum levels diminished over time after cardiac Surgery (Fig. 3A). In contrast, CCL28 increased from baseline at t_7d_ and t_3m_ (Fig. 3B). Not enough patients with pre-existing stroke had blood drawn at 3 months to factor this variable in a longitudinal study. Regression analysis revealed that acute and smoldering inflammation did not correlate with the CCL28 level at all post-surgical times (data not shown). IL-15 level declined in the wake of the surgery, reaching a nadir at 7 days (Fig. 3C).

Perioperative management and dynamics of studied markers.

**Fig. 4:**
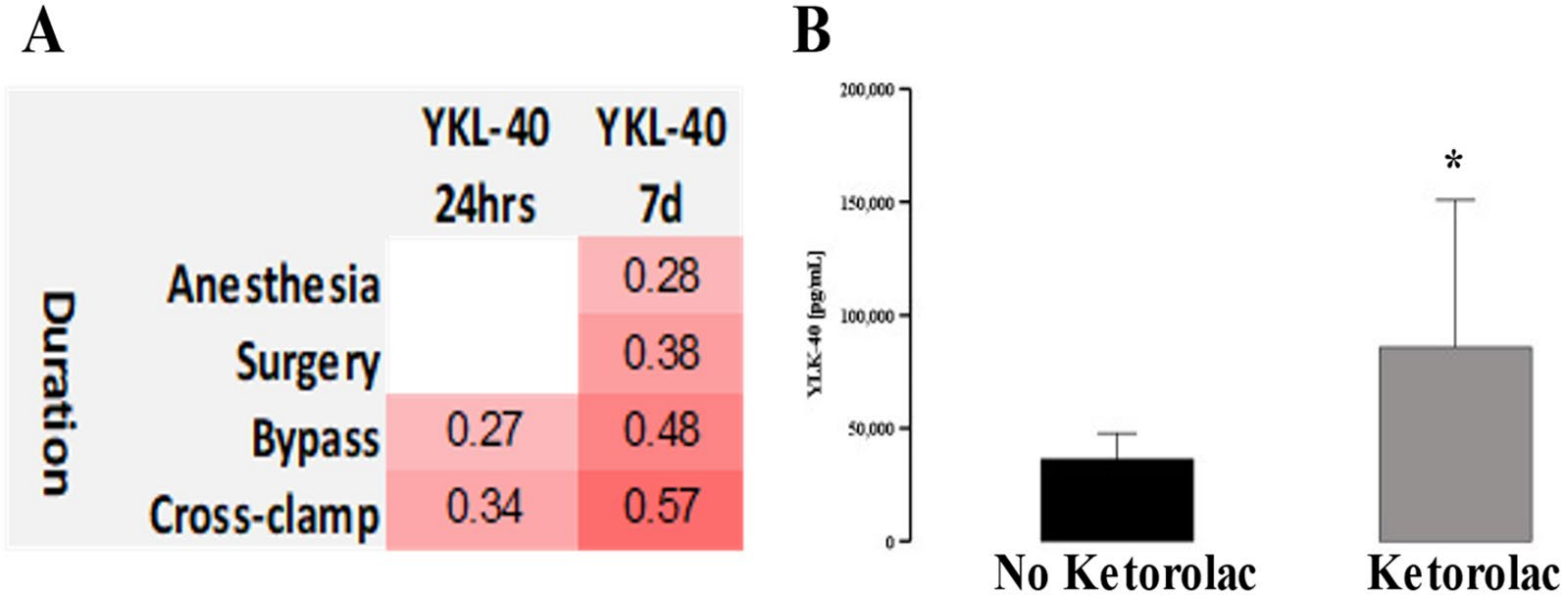
Correlations between perioperative management and significant markers. Serum level of YKL-40 at 24 h and 7 days correlated strongly with several measures for duration of anesthesia **A**. Peri-operative administration of ketorolac correlated with serum level of UKL-40 at 24 h post-operatively **B**. * denotes *p* < 0.05, **p < 0.01, ***p < 0.001

YKL-40 correlated with duration of cardiopulmonary bypass (*r* = 0.27) and cross-clamp (*r* = 0.34). However, a 7 day increase in YKL-40 correlated with the duration of anesthesia, surgery, bypass, and cross-clamp (Fig. 4A). MMP-9 serum level at 3 months correlated with estimated blood loss (*r* = 0.567; *p* = 0.03). There was no difference between all four types of surgery when measured markers were analyzed 24 h and 7 days after the case (data not shown).

Intake of aspirin, steroids, or acetaminophen did not trigger changes in tenascin, YKL-40, and MMP-9. Ketorolac resulted in an increase of serum YKL-40 at 24 h (*p* = 0.01) (Fig. 4B) and borderline at 7 days (*p* = 0.041*one-sided). There was a weak correlation between the dose of ketorolac and YKL increase (*r* = 0.302; *p* = 0.003). There were no significant correlations between intake of opioids and benzodiazepines during surgery, at 24 h, post-ICU admission period, and measured remodeling markers.

### General inflammation is only partially resolved 3 months after surgery

**Fig. 5:**
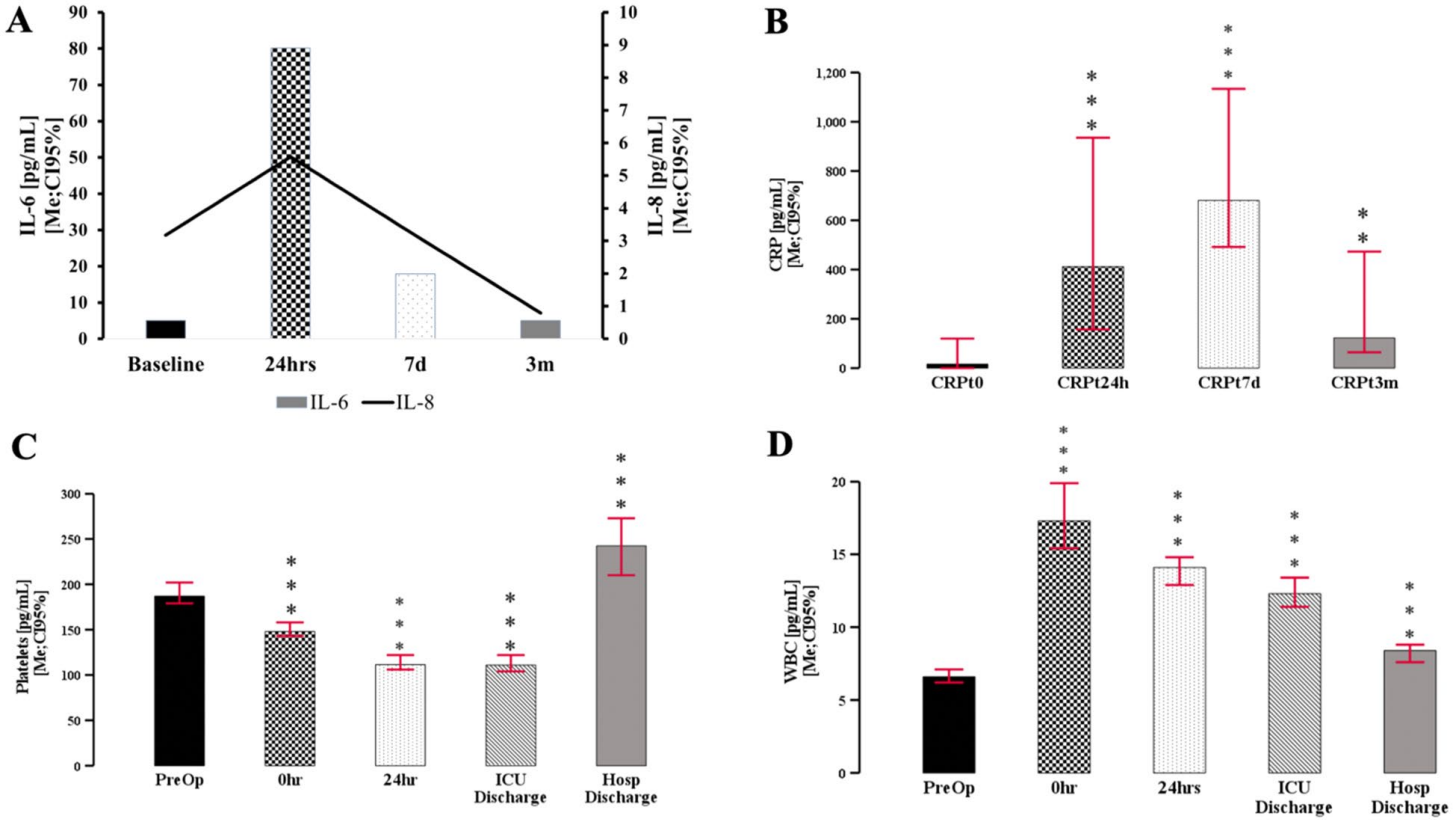
Evolution of the peri-operative inflammatory markers. IL-6 and IL-8 peaked around surgery and reutnred to baselne at 3 months **A**. CRP remained elevated over protracted period of time **B**. Platelets count decline in peri-surgical period but thrombocytosis was appereant at hospital discharge **C**. Concomitantly leukocytosis remained elevated at the discharge as well **D**. * denotes *p* < 0.05, ** *p* < 0.01, ***p < 0.001

IL-6 (F[3;114] = 26; *p* < 0.001) and IL-8 (F[3;120] = 4.5; *p* = 0.011) were significantly elevated in peri-operative period normalized after discharge, while CRP remained elevated (F[3;96] = 24; *p* < 0.001) (Fig. 5A, B). WBC count peaked around admission to the ICU and resolved slowly but not fully at the discharge from the hospital (F[4;138] = 141.7; *p* < 0.0001) (Fig. 5C). Platelets count remained stable after admission to demonstrate a significant increase at the time of discharge (Fig. 5D) (F[4;138] = 71.1; *p* < 0.0001).

### The outcomes and the changes in the level of the measured markers

**Fig. 6:**
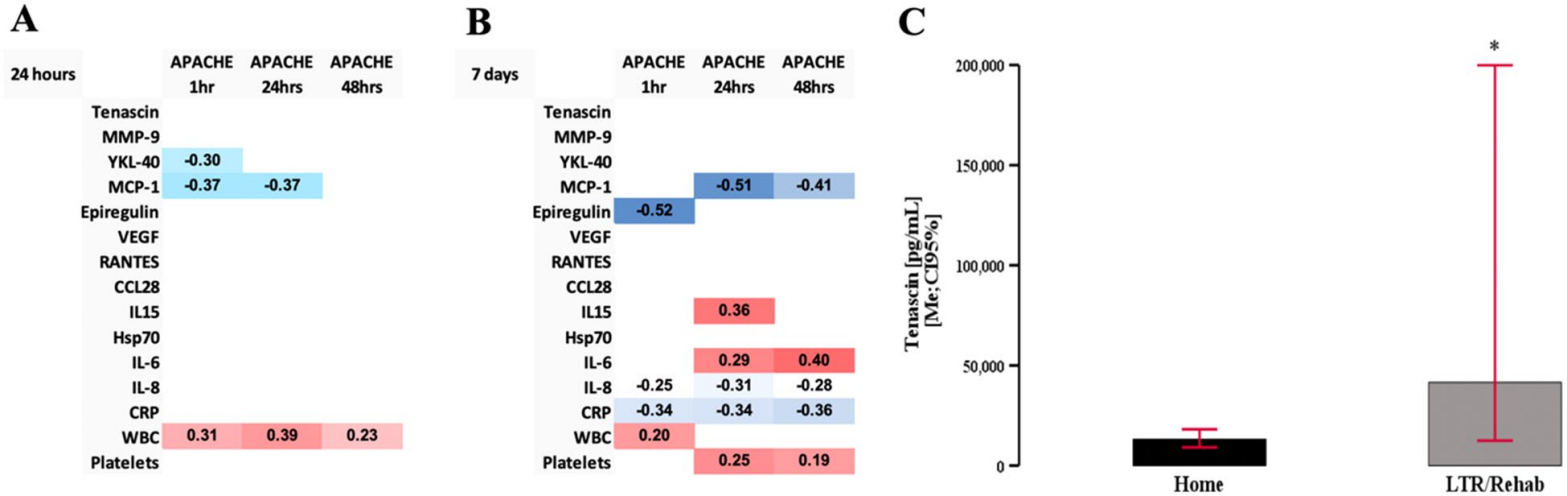
Clinical correlation and measured markers. APACHE II correlated mostly with MCP-1 and WBC 24 h after surgery **A**. Several serum markers correlated with APACHE scores at 7 days, but the correlational matrix was much more heterogenous **B**. Tenascin levels were significantly higher in patients discharged to long-term facilities (LTR) or rehab **C**. * denotes *p* < 0.05

APACHE II at 24 h correlated with tenascin_t24h_ and YLK40_t24_ (Fig. 6A). MCP-1 had a strong correlation with APACHE at all measured time points (Fig. 6A, B). Length of stay correlated with VEGFt_7d_(*r* =  0.46; *p* = 0.04). Disposition at discharge from hospital to home vs. long-term facility was determined by VEGF (VEGF_home_ = 1054.6 ± 317 vs. VEGF_rehab_ = 1544.7 ± 553.6; t[18] = 2.53;*p* = 0.021), and CRP (CRP_home_ = 899.4 ± 608 vs. CRP_rehab_ = 420.6 ± 608; t[37] = 2.12; *p* = 0.041) at 24 h. MCP-1 was significantly lower at 24 h (MCP-1_home_ = 489.1 ± 395 vs. MCP-1_rehab_ = 141.5 ± 167.6; t[33] = 2.089; *p* = 0.044), 7 days (MCP-1_home_ = 640.3 ± 606 vs. MCP-1_rehab_ = 127.5 ± 103.3; t[33] = 2.36; *p* = 0.024) and 3 months (MCP-1_home_ = 775.5 ± 643.9 vs. MCP-1_rehab_ = 175.6 ± 105.6; t[71] = 2.06; *p* = 0.043) if the patient was discharged to a rehabilitation/long-term facility.

Mortality, cerebrovascular accidents, deep venous thrombosis, and acute pulmonary incidence rates in the studied group precluded all statistical analyses. However, tenascin level at baseline determined the outcome at 28 days and 3 months, with patients being admitted to the long-term facility or still in hospital at 28 days (KS[3;124] = 11.1; *p* = 0.011) and 3 months (KS[3;124] = 8.37; *p* = 0.039) (Fig. 6C) had elevated levels of tenascin before surgery.

## Discussion

This is the first study demonstrating the prevalence of the cardiac remodeling serum markers in patients in the convalesce phase of cardiac surgery even after partial resolution of the inflammation. MMP-9, tenascin, and YKL-40 were elevated 3 months since the original surgery, while vascular inflammation and revascularization makers were inconsistently altered. Interestingly, leukocytosis did not resolve and was accompanied by post-operative increased platelet counts and C-reactive levels, suggesting ongoing smoldering inflammation [40]. To date, no other research analyzed the resolution of tissue remodeling longitudinally against the activation of the immune system with follow-up after discharge from the hospital.

Tenascin is frequently released during extracellular remodeling and turnover [27, 70–72]. It is also elevated in exacerbation of congestive heart disease, but no correlation between tenascin and NT-BNP was seen in our study cohort [27]. In addition, our study population consisted of a very heterogenous patient population with some but not a universal representation of patients with decompensated heart failure and severe cardiac disease [27, 73]. Concomitant changes in MMP-9 or YKL-40 may suggest remodeling or ongoing inflammation, yet most of the markers of acute inflammation (IL-6 and IL-8) resolved, while others persevered (CRP, leukocytosis) [33, 52, 66, 74]. However, acute inflammation must evolve into a healing process of tissue [7, 47, 56, 74]. Both markers are elevated in severe ischemic cardiac disease, yet most patients are highly functioning after coronary artery graft bypass surgery or have surgery for non-ischemic process [54, 66, 74]. The increase in markers may suggest ongoing revascularization but most of the revascularization and arterial inflammation markers normalized in our study [5, 6, 9, 26, 34, 51, 52, 66]. Consequently, our study provides evidence that 3 months after heart surgery, tissue remodeling continues to evolve 3 months after heart surgery.

The source of the remodeling markers is unclear, but ongoing myocardial repair and healing is likely. Some of them can be released secondary to mechanical tissue damage. After 3 months of surgery, most of the scarring and tissue repair should be subdued, but the remodeling of the level of tissue structure may continue to progress [12, 50, 56, 72]. Inflammatory cells can release some of the studied markers, but we do not have evidence to support that since IL-6 and IL-8 normalized. The persistent leukocytosis suggests ongoing inflammation, but the white blood cell frequency decreased, while most of the remodeling markers continued to rise in serum. Perivascular tissue is a highly metabolically active organ and a significant source of the remodeling cytokines, but our study cannot determine the source of measured biomarkers [16, 17]. Accelerated fibrosis and arteriosclerosis of the myocardium are linked to increased levels of these markers, but most of our patients had a good recovery after surgery [27, 56]. Alternatively, elevation in tenascin, MMP-9, and YKL-40 represent a nominal healing process [6, 7, 24]. MMP-9 is particularly important to healing as its depletion leads to defective inflammation, worsening the outcome of experimental myocardial infarction [74]. Therefore, the following study should establish the relationship between tenascin and MMP-9 and clinical outcomes considering the pleiotropic nature of these cytokines.

Inflammation in the wake of cardiac surgery was resolved only partially. IL-6 and IL-8 normalized 3 months after the initial peri-operative increase, which is consistent with prior observation [7, 28, 56, 72]. Elevation in C-reactive protein was reported before by our and other groups post-cardiac surgery but also several critical care illnesses [45, 57–59]. It may represent a smoldering inflammation, and simultaneous elevation of CCL28 may underscore that [75]. Leukocytosis and platelets are elevated at discharge from hospital, suggesting that inflammation generally did not resolve entirely except in the most critical aspects. The significance of this finding is twofold. First, unresolved inflammation may result in increased fibrosis, atherosclerosis, and structural changes to the blood vessel wall [3, 18, 20, 21, 57]. These changes will culminate into adverse outcomes, eventually severely mitigating the benefit of surgery. On the positive aspect, most of the markers of the vascular inflammation resolved or were even less pronounced at 3 months as compared to baseline. This suggests that inflammation is systemic and not related to persistently activating endothelium [5, 17, 34]. Alternatively, the leakage of the cytokines from freshly operated and revascularized myocardial endothelium is too obscure to be reflected in whole blood assay.

Perioperative management had a limited impact on the release of the measured markers. This is consistent with the idea that cardiac surgery is significant enough stress elucidating near maximal stress on the homeostasis [1, 7, 28]. Interestingly, peri-operative administration of ketorolac resulted in the elevation of YKL-40 in the peri-operative period but not 3 months. The source of this influence is unclear. Ketorolac is a potent immunomodulator of prostaglandin and prostacyclin with a complex influence on inflammatory response and remodeling [76]. We may demonstrate the relationship between peri-operative intake of ketorolac and some of the remodeling markers, but this observation should be re-evaluated by other centers in a follow-up study, considering no effect of ketorolac on IL-6, the primary inflammatory marker suggesting other mechanisms than inflammation [76, 77].

The clinical impact of the observed changes needs to be established. However, persistent increases in MMP-9 and YKL-40 may result in heart fibrosis [78]. YKL-40 was already elevated in patients before surgery, suggesting ongoing remodeling exacerbated by surgery and returning to normal. YKL-40 is elevated in patients with cardiovascular disease and was reported before [52, 66]. Surgery exacerbated that process peri-operatively, but the level did not normalize after surgery despite addressing the underlying causes of surgery. YKL-40 correlated with duration of surgical procedure and initial APACHE II, an observation concurrent with YKL-40 being inflammatory and heart risk factor [52]. The lack of correlation between MMP-9 may stem from the fact that myocardium injury and subsequent remodeling is the factor, not the inflammation [33].

The study has several strengths. First, the study analyzed the data longitudinally. We controlled for several demographic, peri-operative, and inflammatory markers. The data were coupled with an assessment of the inflammatory markers. The techniques utilized are robust and were used before to assess the measured markers. The results suggest that cardiac remodeling is ongoing 3 months after initial surgery and is accompanied by smoldering inflammation. It remains to be established if the observed changes represent a natural healing process. Alternatively, they may be involved in the increased risk of postoperative adverse events, especially long-term [32, 33, 74, 78].

## Conclusions

The data demonstrated an ongoing extracellular matrix turnover at 3 months, while acute inflammation and vascular remodeling resolved only partially.

## Supplementary Information

**Additional file 1**: Differences at preoperative CCL-28 levels in three pre-existing conditions.

## Data Availability

The data sets used and/or analyzed during the current study are available from the corresponding authors on reasonable request.

## Abbreviations

APACHE: Acute physiologic assessment and chronic health evaluation
IL-15: Interleukin 15
VEGF: Vascular epithelial growth factor
MCP-1: Major chemotaxic factor
AKI: Acute kidney injury
CCI: Charleston comorbidity index
CNSf: Central nervous system failure
COPD: Chronic obstructive pulmonary disease
CRP: C reactive protein
CVA: Cerebrovascular accident
MMP-9: Matrix metallopeptidase 9
RANTES: Regulated upon activation, normal T Cell expressed and presumably secreted
IL-6: Interleukin 6
MODS: Multiple organ dysfunction score
YLK-40: Chitinase-3-like protein 1
