# Supplementary material for: Concomitant elevated serum levels of tenascin, MMP-9 and YKL-40, suggest ongoing remodeling of the heart up to 3 months after cardiac surgery after normalization of the revascularization markers
Source: Eur J Med Res. 2022 Oct 21;27:208. doi: 10.1186/s40001-022-00831-8 (PMC9585873; doi:10.1186/s40001-022-00831-8)

# Additional Files

**Supplemental Material #1.**

Differences at preoperative CCL-28 levels in three pre-existing conditions


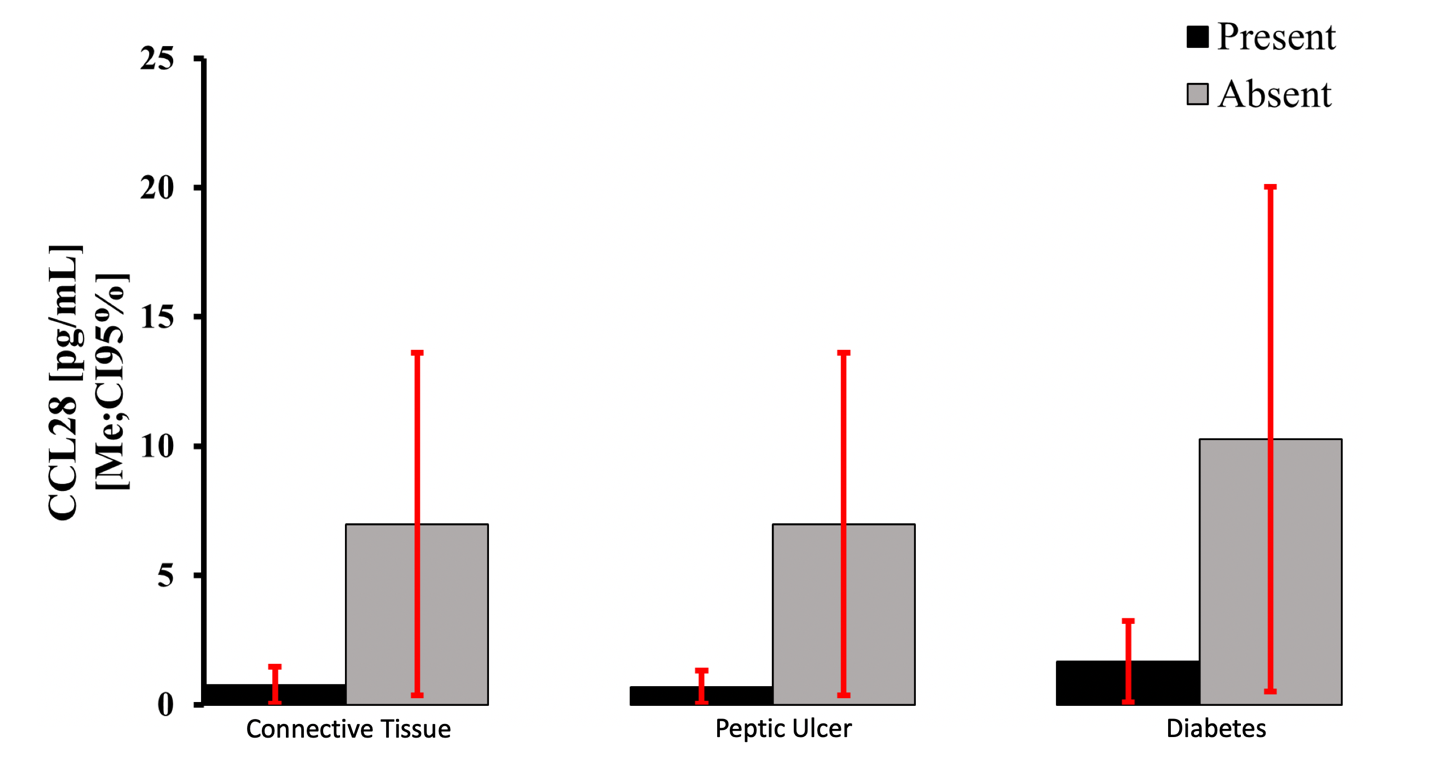

Supplement: Supplementary file 1 — Additional file 1: Differences at preoperative CCL-28 levels in three pre-existing conditions. [file 40001_2022_831_MOESM1_ESM.docx]
